# The impact of REM sleep loss on human brain connectivity

**DOI:** 10.1038/s41398-024-02985-x

**Published:** 2024-07-02

**Authors:** Tianqi Di, Libo Zhang, Shiqiu Meng, Wangyue Liu, Yang Guo, Enyu Zheng, Chao Xie, Shitong Xiang, Tianye Jia, Lin Lu, Yan Sun, Jie Shi

**Affiliations:** 1 Department of Pharmacology, School of Basic Medical Sciences, National Institute on Drug Dependence, Peking University, Beijing, 100191 China; 2 Beijing Key Laboratory of Drug Dependence, Peking University, Beijing, 100191 China; 3 Shenzhen Key Laboratory for Drug Addiction and Medication Safety, Shenzhen Public Service Platform for Clinical Application of Medical Imaging, Department of Ultrasound, Peking University Shenzhen Hospital, Shenzhen-PKU-HKUST Medical Center, Shenzhen, 518036 China; 4 Institute of Science and Technology for Brain-Inspired Intelligence, Fudan University, Shanghai, 200433 China; 5 The Key Laboratory for Neuroscience of the Ministry of Education and Health, Peking University, Beijing, 100191 China; 6 The State Key Laboratory of Natural and Biomimetic Drugs, Peking University, Beijing, 100191 China; 7 Henan Collaborative Innovation Center of Prevention and Treatment of Mental Disorder, Xinxiang, 453000 China

**Keywords:** Human behaviour, Neuroscience, Predictive markers

## Abstract

Brain function is vulnerable to the consequences of inadequate sleep, an adverse trend that is increasingly prevalent. The REM sleep phase has been implicated in coordinating various brain structures and is hypothesized to have potential links to brain variability. However, traditional imaging research have encountered challenges in attributing specific brain region activity to REM sleep, remained understudied at the whole-brain connectivity level. Through the spilt-night paradigm, distinct patterns of REM sleep phases were observed among the full-night sleep group (*n* = 36), the early-night deprivation group (*n* = 41), and the late-night deprivation group (*n* = 36). We employed connectome-based predictive modeling (CPM) to delineate the effects of REM sleep deprivation on the functional connectivity of the brain (REM connectome) during its resting state. The REM sleep-brain connectome was characterized by stronger connectivity within the default mode network (DMN) and between the DMN and visual networks, while fewer predictive edges were observed. Notably, connections such as those between the cingulo-opercular network (CON) and the auditory network, as well as between the subcortex and visual networks, also made significant contributions. These findings elucidate the neural signatures of REM sleep loss and reveal common connectivity patterns across individuals, validated at the group level.

## Introduction

Sleep deprivation, influenced by lifestyle factors such as shift work, psychosocial stress, and excessive electronic media use, poses a significant public health challenge in modern society [1]. Research indicates that more than a third of adults fail to obtain sufficient sleep regularly [2]. Inadequate sleep has been associated with a range of negative outcomes, from disruptions in a wide range of emotional processes [3], though to an increased risk of metabolic disease and obesity [4]. Importantly, there is a close association between sleep loss and functional impairments in our brain [5–9]. However, these studies have overlooked the relationship between inadequate sleep and the dynamic reorganization of the brain.

Interestingly, sleep deprivation encompassing the loss of two distinct sleep phases: rapid eye movement (REM) sleep and non-rapid eye movement (NREM) sleep [10]. The distribution of these sleep phases varies between early and late-night sleep. In the early-night period, NREM sleep predominates, whereas REM sleep takes precedence during the later stages of the night [11, 12]. During REM sleep, the brain not only maintains energy balance but also clears metabolic byproducts and waste, crucial for sustaining optimal brain function [13, 14]. Some research found that REM sleep contributes significantly to emotional memory and creative thinking, compared to NREM sleep [15, 16]. Furthermore, the REM phase has also been linked to the coordination of various brain structures. Firstly, REM ponto-geniculo-occipital (PGO) waves originate from the brainstem and propagate throughout the entire brain [17]. Secondly, inhibition of theta waves, which are characteristic of REM sleep, can impair hippocampus-dependent memory consolidation [18]. Additionally, REM sleep promotes selective reinforcement or suppression of dendritic spines in the neocortex [19]. Suggesting REM sleep plays a critical role in human brain function, however, research on the brain mechanisms of how REM sleep affects brain function remains limited. Therefore, we aimed to investigate the relationship between individual differences in REM sleep and functional connectivity of the brain’s resting state. Moreover, given the challenges in selectively manipulating REM sleep without disrupting sleep structure in humans, the role of REM sleep loss on functional connectivity remains far from clear.

Neuroimaging studies revealed disturbed functional activity and connectivity in executive control regions, hippocampal and amygdala circuits, default mode network (DMN), attention and salience network of brain respectively after insufficient sleep [20–22]. While our brain constitutes a complex structure comprising numerous large-scale networks, the dynamic nature of the intricate interconnections within the brain supports its overall function [23]. It is necessary to identify the whole brain patterns of generate brain-REM sleep model. Connectome-based predictive modeling (CPM) is a computational framework to investigate and predict individual differences in brain connectivity patterns based on whole-brain functional connectivity (FC) data [24]. Compared to traditional regression model, CPM can prevent overfitting by defining and validating in independent samples, thus leading to more accurate effect sizes and enhancing the generalizability of discoveries [25], it is a powerful tool for identifying REM sleep connectome.

Two pivotal inquiries await elucidation. The initial query revolves around delineating the specific brain networks associated with REM sleep, integrating resting-state fMRI data across diverse distributions of REM sleep stages. The subsequent inquiry delves into whether the lack of REM sleep, encompassing both early- and late-night sleep, exerts an impact on REM-brain networks compared with a sleep rested night, particularly considering the dominance of REM sleep during the late-night. To answer these questions, we utilized the split-night paradigm and a between-subject design, inducing differing levels of REM sleep loss through two half-night sleep and one full-night sleep conditions, across healthy adults. This segmentation enabled us to explore potential variations in REM sleep characteristics between these two-time frames and served as a baseline for comparing the effects of various types of sleep loss on REM sleep. We utilized two key metrics, duration and proportion, to quantify the total time spent and the percentage of total sleep time occupied by REM sleep during the sleep cycle, respectively. These metrics are commonly utilized to assess sleep quality, variations in sleep patterns, and pathological features of sleep disorders [26]. Subsequently, we applied CPM to discern REM sleep-associated brain networks on a large-scale level. Moreover, we investigated whether disparities in the lack of REM sleep contributed to the interactions within the CPM-derived networks among groups.

## Method

### Participants

A total of 113 right-handed healthy adults were recruited in multiple universities in Beijing (Peking University, Tsinghua University, Beijing University of Aeronautics and Astronautics, Beijing Forestry University, Beijing Normal University, China Agricultural University) for our study and randomly assigned to one of three groups (See Table 1): 1) the late-night sleep deprivation group (Late-deprivation, sleep from 23:00 to 03:30, *n* = 41); 2) the early-night sleep deprivation group (Early-deprivation, sleep from 03:00 to 07:30, *n* = 36); 3) the full-night sleep group (FS, sleep from 23:00 to 8:00, *n* = 36). The following morning, all participants underwent rs-fMRI scans after awakening and after overcoming 30 min of sleep inertia (from 08:00 to 10:00). If a subject’s sleep latency exceeded 30 min during the sleep monitoring period, or if there was a single awakening lasting longer than 30 min, the experiment was terminated. Similarly, if a subject fell asleep during the sleep restriction period, the experiment was concluded, and the subject was escorted from the laboratory by the experimenter.

**Table 1:** Demographic Information and sleep characteristics.

	Early-Deprivation Group (*n* = 41)	Late-Deprivation Group (*n* = 36)	Full-night Sleep Group (*n* = 36)	*P*
Age (years)	23.00 ± 2.53	22.88 ± 2.44	22.78 ± 4.44	0.957
Female (n, %)	20 (48.78)	13 (36.11)	21 (58.33)	0.291
BMI	22.23 ± 2.95	21.93 ± 2.43	21.35 ± 4.45	0.552
PSQI Score	3.05 ± 1.51	3.44 ± 1.44	3.36 ± 1.57	0.484
MEQ Score	51.25 ± 5.74	51.14 ± 5.66	48.17 ± 9.97	0.130
SAS Score	29.14 ± 3.56	28.03 ± 3.89	28.97 ± 4.17	0.414
SDS Score	30.22 ± 6.11	27.89 ± 5.40	29.22 ± 6.06	0.255
Sleep Parameters in Adaption Night				
TST (min)	442.66 ± 57.33	449.75 ± 38.31	443.99 ± 37.99	0.810
SL (min)	13.95 ± 12.96	9.62 ± 5.99	9.55 ± 4.93	0.070
WASO (min)	31.65 ± 32.65	31.50 ± 29.61	31.41 ± 20.43	0.991
SE (%)	0.93 ± 0.15	0.93 ± 0.06	0.92 ± 0.07	0.450
N1 (%)	7.02 ± 4.65	7.67 ± 3.83	7.36 ± 4.89	0.871
N2 (%)	49.05 ± 9.46	50.49 ± 7.69	47.89 ± 9.26	0.511
N3 (%)	21.82 ± 9.04	20.06 ± 7.72	23.69 ± 8.52	0.243
REM (%)	21.84 ± 4.72	21.81 ± 4.38	21.92 ± 9.48	0.980
Sleep Parameters in Sleep Manipulation Night				
TST (min)	251.22 ± 26.00	251.86 ± 26.69	452.93 ± 37.54	< 0.001***
SL (min)	4.86 ± 3.33	9.80 ± 13.31	7.81 ± 6.58	0.046*
WASO (min)	6.45 ± 7.40	10.29 ± 17.59	23.31 ± 23.16	< 0.001***
SE (%)	0.96 ± 0.03	0.93 ± 0.08	0.92 ± 0.07	0.051
N1 (%)	4.50 ± 2.64	6.18 ± 4.48	6.05 ± 5.28	0.177
N2 (%)	45.73 ± 9.11	51.98 ± 9.10	50.82 ± 9.79	0.013*
N3 (%)	25.79 ± 6.70	27.14 ± 9.16	19.73 ± 7.11	< 0.001***
REM (%)	24.41 ± 6.56	15.18 ± 5.31	22.56 ± 6.32	< 0.001***

*BMI* body mass index, *PSQI* Pittsburgh Sleep Quality Index, *MEQ* Horne-Ostberg Morningness-Eveningness Questionnaire, *SAS* Self-Rating Anxiety Scale, *SDS* Self-Rating Depression Scale, *TST* total sleep time, *SL* sleep latency, *WASO* wake after sleep onset, *SE* sleep efficiency, *REM* rapid eye movement, *%* percentage

Values are presented as mean ± SD.

*p **<** 0.05, ***p < 0.001.

Their sleep patterns were confirmed through a seven-day sleep diary and the use of a sleep actigraphy (Spectrum and pro, Philips Respironics, Inc., Murrysville, PA, Oregon). All of the participants were instructed to refrain from drug, alcohol, and caffeine consumption for 48 h before the study. Participants reported an average sleep duration of 7-8 h per day had no history of psychiatric or neurological illness, nor did they engage in illegal drug use. Additionally, they were free from MRI contraindications. Female participants reported regular menstrual cycles and were not using oral contraceptives before or during the study. The experimental procedures received ethical approval from the Ethics Committee at Peking University (The Code of Ethics: IRB00001052-23141). Prior to their involvement, all individuals provided written informed consent and were compensated for their participation.

### Polysomnographic

In the laboratory setting, Somte polysomnographic (PSG) mobile recording system (Grael, Compumedics Inc., Charlotte, NC) were obtained for all subjects over two consecutive nights. The first night served as an adaptation night to mitigate first-night effects and exclude the influence of sleep disorders, ensuring participants acclimated to the experimental environment. The second night constituted the formal sleep experimental night, dedicated to the comprehensive collection of sleep data. PSG recordings of sleep encompassed the measurement of various physiological parameters. Electroencephalography (EEG) channels were strategically placed at F3, F4, C3, C4, O1, and O2, with reference to the contralateral mastoid, following the International 10–20 system. Bilateral electromyography (EOG) and submental is electrocardiography (EMG) data were concurrently recorded.

Sleep stages were manually scored in 30-second epochs using the standard criteria for polysomnographic sleep recording, as outlined by the American Academy of Sleep Medicine (AASM) guidelines. Two independent sleep technicians, who were unaware of the group assignments, performed the scoring.

### Spilt-night procedure

Participants were divided into three groups, with two groups following a spilt-night sleep paradigm that involved Late-deprivation and Early-deprivation groups. Both groups were allocated a total sleep opportunity of 4.5 h [12, 27]. Participants in Late-deprivation group slept from 23:00 to 03:30 and remained awake from 03:30 to 07:30. This schedule was designed to ensure that participants experienced predominantly NREM sleep in the first half of the night and stayed awake during the second half. Participants in Early-deprivation group stayed awake from 23:00 to 03:00 and slept from 03:00 to 07:30. This schedule was intended to ensure that participants experienced predominantly REM sleep during the latter half of the night, remaining awake in the first half. While awake, participants were allowed to engage in quiet activities such as reading printed books or paper materials and walking indoors. They were also permitted to drink water in moderation. These activities were chosen to maintain a low level of physical and cognitive stimulation.

### Rs-fMRI preprocess

During the scanning procedure, participants were instructed to keep their eyes closed throughout the entire session and make every effort to minimize head movement. Rs-fMRI data were acquired using a 3.0-T GE Discovery MR750 system (General Electric Medical System, Milwaukee, WI, USA). A total of 33 axial slices were collected with a slice thickness of 4.2 mm and no gap between slices. The 8-min images used an echo-planar imaging (EPI) sequence with a repetition time (TR) of 2 s. The dataset consisted of 240 time points with an echo time of 30 ms and a flip angle of 90°, and an in-plane resolution of 3.5 × 3.5 mm² with a field of view measuring 224 × 224 × 64 mm³. For structural reference, a T1-weighted image was obtained for each participant with a voxel size of 1 × 1 × 1 mm³ and 192 slices (TR = 6.7 ms, echo time = Min Full, flip angle = 12°, resolution matrix = 256 mm × 256 mm, thickness = 1.0 mm).

Data preprocessing was performed using Statistical Parametric Mapping (SPM12, https://www.fil.ion.ucl.ac.uk/spm/software/spm12/) and Data Processing and Analysis for Brain Imaging (DPABI) toolboxes [28] running on MATLAB. The first ten volumes of each run were discarded to allow for MRI T1 equilibration. Subsequently, all functional volumes were realigned to the mean image and co-registered to anatomical images with affine transformations, conducted separately for each volunteer. Anatomical scans underwent DARTEL normalization to Montreal Neurological Institute (MNI)-space following segmentation. The same transformation was applied to co-registered functional volumes, employing a small smoothing kernel with a Full Width at Half Maximum of 6 × 6 × 6 mm. Any images exhibiting an average head motion displacement exceeding 3.0 mm or rotational movement beyond 3.0° were systematically excluded from further analysis.

### Construction of large-scale functional networks

The brain of each subject was partitioned into 227 cortical and subcortical regions using the Power template [29] (Table S1). These nodes were categorized into 10 brain networks, including the default mode network (DMN), the visual network (VIS), the frontal-parietal network (FPN), the dorsal attention network (DAN), the ventral attention network (VAN), the salience network (SAN), the cingulo-opercular network (CON), the auditory network (AUD), the sensorimotor dorsal network (SMN) and the subcortical network (SUB) [30, 31].

### Connectome-based predictive modeling (CPM)

To explore the neural network connectivity patterns related to sleep loss, we employed Connectome-based Predictive Modeling (CPM) using resting-state functional magnetic resonance imaging data. In the CPM model, sleep data (the duration of REM sleep) and whole-brain connectivity matrices were utilized as inputs to construct a model of brain behavior. For each individual, the positive edge weights (edges with positive connection strengths) and negative edge weights (edges with negative connection strengths) are summed separately to obtain the total positive and negative edge weights, then used as individual features inputted into predictive model. Consistent with recommendations for predictive modeling on moderate-sized neuroimaging samples [32], the current analysis employs Leave-One-Out Cross-Validation (LOOCV).

### Network connectivity

For each subject, the time series for each region of interest (ROI) was extracted from the preprocessed data. Functional connectivity matrices were then constructed by calculating the mean time series of all 227 regions and computing Pearson’s correlation coefficients for each pair of regions. The resulting correlation coefficients were transformed into z‐values using Fisher’s Z transformation.

## Results

### Timing and sleep manipulation effects on REM sleep patterns

**Fig. 1:**
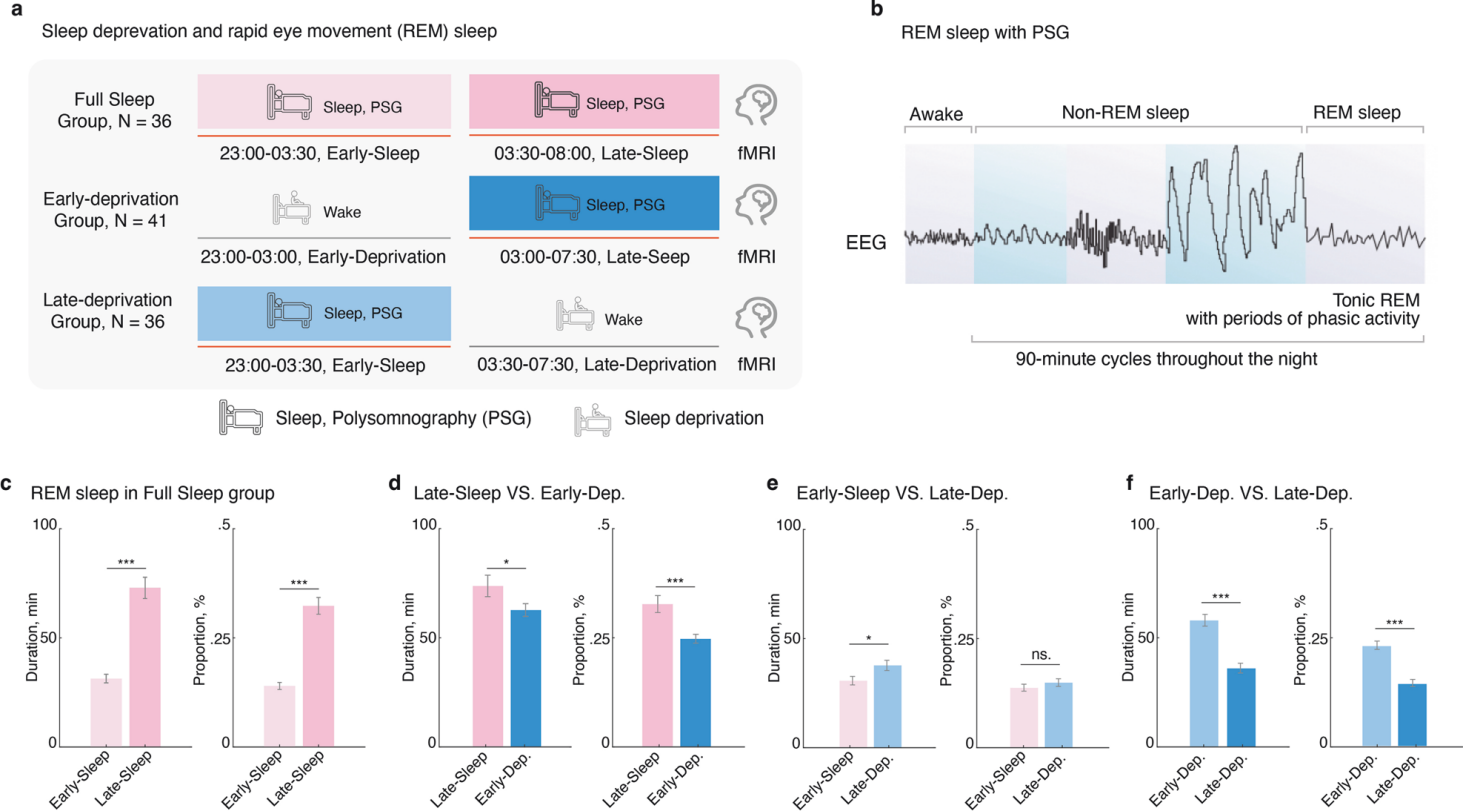
Late-night REM sleep, but not early-night REM sleep, maintains optimal REM sleep patterns. **a** Experimental timeline for manipulating REM sleep loss procedure. **b** Diagram for the sleep progression from awake to non-rapid eye movement (NREM), and rapid eye movement (REM) sleep, along with the changes in electroencephalography (EEG), was tracked using Polysomnography (PSG) over a 90-minute cycle. **c** Comparison of REM phase duration and percentage between early-night and late-night sleep in the Full-Sleep Group, revealing higher values during early-night sleep. **d** Decreased REM phase duration and percentage in the Early-Deprivation Group compared to late-night sleep in the Full-Sleep Group. **e** Increased REM phase duration and percentage in the Late-Deprivation Group compared to early-night sleep in the Full-Sleep Group. **f** Significantly better REM phase duration and percentage in the Early-Deprivation Group compared to the Late-Deprivation Group. *p < 0.05; ***p < 0.001. ns. Not Significant. Data are presented as the mean ± SEM.

To investigate whether the variability of different REM sleep phase, we conducted a segment analysis on the REM sleep patterns within the full-night sleep group (FS). Specifically, we segmented the full night’s sleep into two distinct periods: early-night (23:00-03:30, early-FS) and late-night (03:30-08:00, late-FS) (Fig. 1a). We found that during late-night sleep, both the duration (*n* = 33, *t* = 8.26, Cohen *d* = 1.40, *P*_*two-tailed*_ = 1.93e-09, (Fig. 1b) and proportion (*n* = 33, *t* = 8.76, Cohen *d* = 1.55, *P*_*two-tailed*_ = 5.15e-10, Fig. 1c) of REM sleep significantly increased compared to early-night sleep. This finding underscores the importance of integrating sleep timing to fully elucidate its impact on REM sleep patterns. Next, we compared the changes in REM sleep between the early-FS group and the late- deprivation group, as well as between the late-FS group and the early- deprivation group respectively (Fig. 1d).

We found that compared to the late-FS group (*n* = 36), early-deprivation group (*n* = 41) significantly decreased duration (*t* = −2.02, Cohen *d* = −0.24, *P*_*two-tailed*_ = 0.046) and proportion (*t* = −3.82, Cohen *d* = 0.67, *P*_*two-tailed*_ = 2.81e-04) of the REM sleep. In contrast, compared to the early-FS group, late-deprivation group (*n* = 36) only significantly decreased the duration of REM sleep (*t* = −2.27, Cohen *d* = −0.29, *P*_*two-tailed*_ = 0.026), but not the proportion (*t* = −0.99, Cohen *d* = −0.12, *P*_*two-tailed*_ = 0.32). These results suggest that both early and late deprivation of sleep have a significant impact on REM sleep. However, we have to voluntarily sacrifice sleep to manage daily activities. Hence, it is crucial to compare the differing effects of two types of sleep deprivation on REM sleep to support our decisions regarding sleep management. We found that early-deprivation group has significantly better REM sleep pattern, compared to late-deprivation group (Duration: *n* = 77, *t* = 6.17, Cohen *d* = 0.70, *P*_*two-tailed*_ = 3.19e-08; Proportion, *n* = 77, *t* = 6.80, Cohen *d* = 0.78, *P*_*two-tailed*_ = 2.20e-09), suggesting that the early-deprivation group exhibits a more favorable REM sleep pattern compared to the late-deprivation group.

### Connectome-based prediction of the REM sleep

**Fig. 2:**
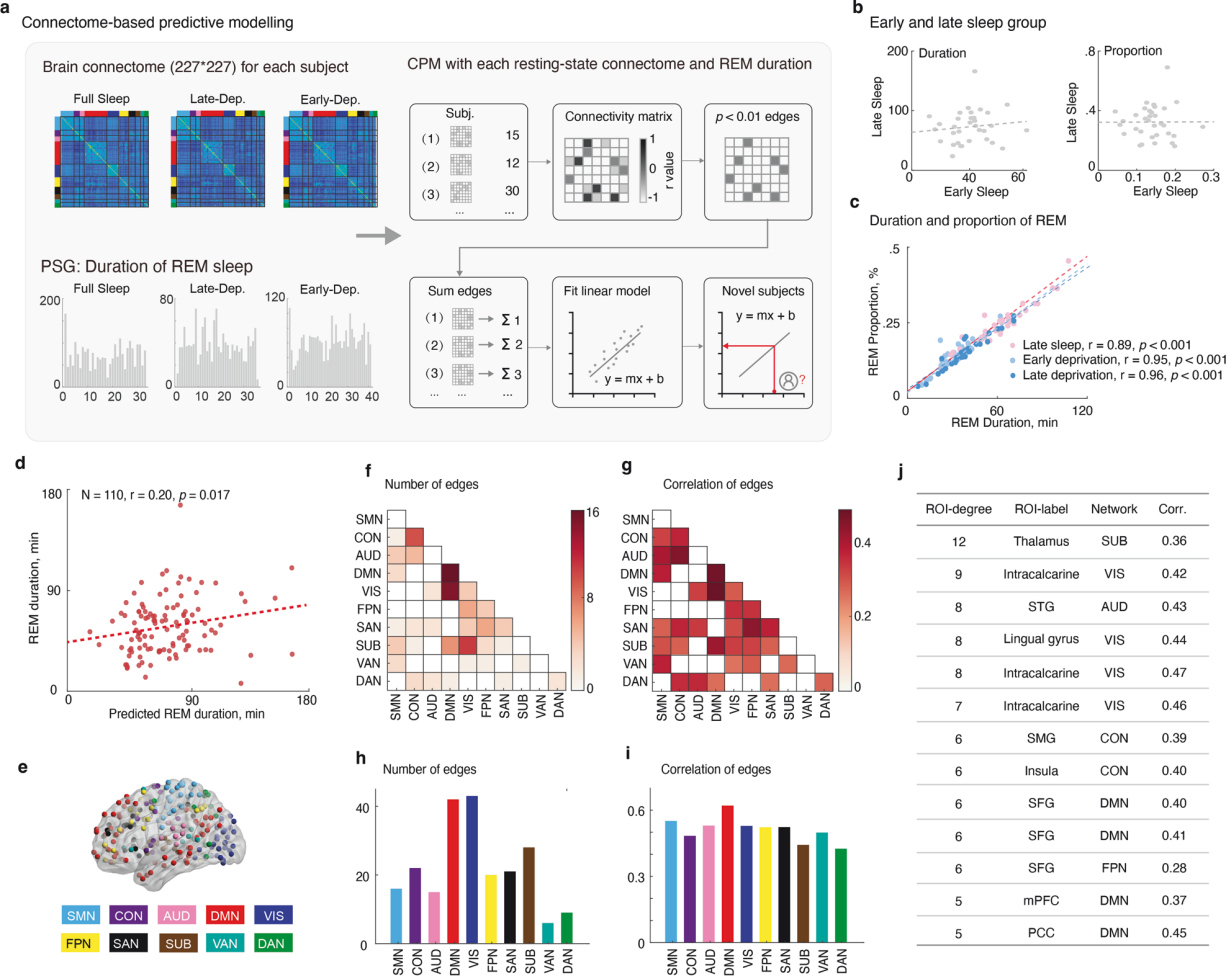
Schematic the analyses and results of REM sleep connectomes. **a** A large-scale functional connectivity matrix, consisting of 227×277 nodes for each subject, was utilized in connectome-based predictive modeling (CPM) to generate a brain-REM sleep model at the whole-brain level. **b** Illustrating no correlation between REM sleep during late-night sleep and early-night sleep. **c** Demonstrating a high correlation between the duration and proportion of REM sleep among groups. **d** Identifying the positive network that significantly predicts REM sleep in the CPM model. **e** Employing a network-based analysis to detect networks with significant nominal changes in functional connectivity matrices: somatosensory/motor network (SMN), cingulo-opercular network (CON), auditory network (AUD), default mode network (DMN), visual network (VIS), fronto-parietal network (FPN), salience network (SAN), subcortical network (SUB), ventral attention network (VAN), and dorsal attention network (DAN). **f**–**i** Showing a notable increase in the number (**f**, **h**), particularly in the DMN-VIS and SUB-VIS connections, and correlations (**g**, **i**), particularly in the DMN and CON, of edges in the REM connectome. Revealing the significant roles played by the majority of brain networks in predicting the connectomes. **j** Highlighting the thalamus, visual cortex, and auditory cortex as having the most edges and making high contributions in terms of regional edges and regional correlation.

We used the connectome-based predictive modeling (CPM) to generate brain-REM sleep model from whole-brain functional connectivity data, which was termed as “REM connectomes”. To enhance the statistic power, we combined the behavioral data of REM sleep from three groups: late-FS group, late-deprivation group, early-deprivation group. We retained the late-FS group for two reasons instead of the early-FS. First, previous analyses had demonstrated that the late-FS group exhibits a significantly longer duration and higher proportion of REM sleep; Second, there was no correlation between REM sleep in the late-FS and the early-FS group (Duration: *n* = 33, *r* = 0.12, *P*_*two-tailed*_ = 0.51; Proportion: *n* = 33, *r* = 0.004, *P*_*two-tailed*_ = 0.98). These findings suggest that the late-FS group better reflects individual differences in REM sleep. Besides, we found that the duration and proportion of REM sleep has a high correlation to each other in three groups (late-night sleep group: *n* = 33, *r* = 0.89, *P*_*two-tailed*_ = 2.58e-12; early-deprivation group: *n* = 41, *r* = 0.95, *P*_*two-tailed*_ = 2.74e-21; late-deprivation group: *n* = 36, *r* = 0.96, *P*_*two-tailed*_ = 2.72e-20). Therefore, we only used the duration of REM sleep in brain-behavior modelling analysis. Using the CPM model, we found that the positive network significantly predicts the REM sleep (*n* = 110, *r* = 0.20, *P*_*one-tailed*_ = 0.017, Fig. 2a. as the predictive effect is unidirectional, the reported *P* value here is one-tailed).

### Multi-level characterization of REM sleep connectome

To comprehensively understand the underlying neural mechanism of the REM sleep connectome, we conducted a multi-level characterization analysis, encompassing brain networks, large-scale networks, and specific brain regions. Additionally, previous studies employing CPM-based characterization typically relied on edge counts in predictive models. However, since the predictive algorithm of CPM is based on linear regression [25], we also utilized correlation to assess the importance of predictive features.

The CPM connectome primarily resides within certain brain networks (variance of normalized predictive edges = 0.22), notably the default mode network (DMN-DMN) and the cingulo-opercular network (CON-CON), as well as between the default mode network and the visual network (DMN-VIS), and between the subcortical network and the visual network (SUB-VIS) (Fig. 2b). Regarding network contribution, the majority of brain networks play significant roles in prediction, rather than just a few networks (variance of normalized correlation = 0.13, Fig. 2c). Notably, the DMN-DMN and DMN-VIS also showed an important contribution. However, networks with fewer predictive edges also made important contributions, such as CON-Auditory, SUB-VIS networks. Details are provided in the supplementary materials Table S2a.

Next, we characterize the importance and contribution of the ten large-scale networks in the REM sleep connectome (Table S2b). We found that the DMN, VIS and SUB networks has the predominant predictive edges (51%, Fig. 2d), which was consistent with the pattern of brain networks (variance of normalized predictive edges = 0.28). However, in terms of large-scale network contribution, the prediction contribution was widely distributed across the ten networks (variance of normalized correlation = 0.09, Fig. 2e). We also found that the DMN, sensory motor network (SMN), VIS and Auditory networks have an important role in the predictive model (Table S3).

Last, we checked the regional importance and contribution in the REM sleep connectome. In terms of regional edges and regional correlation, we found that the two measures have a high similarity (Table S4. *n* = 227, *r* = 0.79, *P*_*two-tailed*_ = 6.17e-50), suggesting that the role of brain regions at a small scale is remarkably consistent. Specifically, we found that the thalamus and visual, and auditory cortex had the most edges and high contribution, such as calcarine, lingual gyrus, and superior temporal gyrus (STG) (Fig. 2f).

### Effects of sleep deprivation on REM sleep connectome

In the above analyses, we identified a whole-brain REM sleep connectome, which was consisted of DMN, SUB and VIS networks. Furthermore, we last investigated whether half-night sleep deprivation affects the REM sleep connectome.

**Fig. 3:**
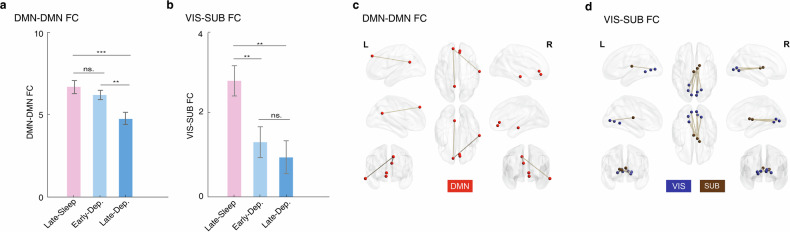
Late-night sleep deprivation leads to greater reduction in REM sleep connectome. **a** Demonstrates that the DMN-DMN connectivity in the late-deprivation group was the lowest among the groups. **b** Illustrates that the VIS-SUB connectivity in the late-sleep group was significantly higher than in the early-deprivation group and late-deprivation group. **c** Diagrams for six connections indicating significant group differences in the DMN-DMN network. **d** Displays diagrams for eight connections indicating significant group differences in the VIS-SUB network. **p < 0.01; ***p < 0.001. ns. Not Significant. Data are presented as the mean ± SEM.

Among the 32 brain networks of REM sleep connectome, only two brain networks showed significant group difference between late-FS group, late-deprivation group, early-deprivation group after multiple correction (Table S5. DMN-DMN: F(2,107) = 8.10, *P*_*two-tailed*_ = 5.29e-04; VIS-Subcortex: F(2,107) = 6.19, *P*_*two-tailed*_ = 0.003). We further found that the DMN-DMN connectivity of late-deprivation group was significantly lower than late-FS group (*n* = 69, *t* = 3.55, Cohen *d* = 0.43, *P*_*two-tailed*_ = 7.12e-04) and early-deprivation group (*n* = 77, *t* = 3.18, Cohen *d* = 0.36, *P*_*two-tailed*_ = 0.002) (Fig. 3a). In contrast, the Visual-Subcortex (VIS-SUB) connectivity of late-FS group was significantly higher than early-deprivation group (*n* = 74, *t* = 2.80, Cohen *d* = 0.43, *P*_*two-tailed*_ = 0.007) and late-deprivation group (*n* = 69, *t* = 2.80, Cohen *d* = 0.43, *P*_*two-tailed*_ = 0.007) (Fig. 3b).

Next, we further compared the REM sleep related connection of the DMN-DMN and VIS-SUB networks between the three groups. For the DMN-DMN network, we found six connections showed significant group difference (Table S6, Fig. 3c), such as connection between medial frontal cortex (mPFC) and paracingulate (F(2,107) = 4.01, *P*_*two-tailed*_ = 0.029) and connection between superior frontal gyrus (SFG) and the posterior cingulate cortex (PCC) ((F(2,107) = 3.11, *P*_*two-tailed*_ = 0.048). Similarly, the connection of VIS-SUB of late-deprivation group was significantly lower than late-FS group (Table S7). For the VIS-SUB network, we found eight connection with significant group difference (Table S8), especially the connection between thalamus and lingual gyrus (F(2,107) = 7.68, *P*_*two-tailed*_ = 6.61e-04) and the connection between thalamus and calcarine (F(2,107) = 6.34, *P*_*two-tailed*_ = 0.003). Similarly, the connection of VIS-SUB network of late-FS group was significantly higher than early-deprivation group (Table S9).

## Discussion

The present study delved into the effect of REM sleep loss on resting brain functional connectivity at the multi-level. Through the CPM analysis, we discerned the effects of REM sleep on the functional connectivity of the brain (REM connectome) during its resting state across various conditions of REM sleep loss. Notably, the REM connectome predominantly manifested within the DMN and CON network, as well as between the DMN and VIS network, and between the SUB and VIS network. Moreover, at the network level, the DMN, VIS, and SUB networks exhibited greater contributions to the REM connectome, while at the regional level, the calcarine, lingual gyrus, and superior temporal gyrus displayed heightened involvement in the REM connectome.

Interestingly, we observed that the thalamus exhibited the highest degree centrality and made a significant contribution to the REM connectome. Additionally, the subcortical networks, to which the thalamus belongs, displayed the third most prominent predictive edges. During REM sleep, the thalamus acts as a relay station for sensory information, transmitting signals from the environment to the cerebral cortex. It is involved in regulating the transition between different sleep stages, including the onset and termination of REM sleep cycles [33]. Moreover, we also found that the insula was with high regional degree and contribution, which was plays a crucial role in the interaction between internal and external perception [34]. Given that cortical and thalamocortical activity is highly state dependent [35], the subcortical network that involved in regulation of arousal and sleep [36], it is possible that lack of REM sleep may influence emotion expression and cognitive behavior through the thalamus and its network [37]. Evidence from deep brain stimulation research supported the heterogeneity REM sleep is not limited to cortical activity, but is also manifested by anterothalamic and thalamocortical synchronization [38]. Corroborating this possibility, previous studies have shown that the mental processes such as episode memory, cognition and emotion, whose pathological changes are closely correlated with the occurrence of psychiatric disorders such as posttraumatic stress disorder and addiction [39–41], are more predisposed to be processed during the REM phase [6, 42].While both types of sleep deprivation affect REM sleep, when faced with the necessity of sleep loss, prioritizing sleep during the latter part of the night may be a preferable choice for maintaining optimal REM sleep patterns.

The DMN is distinguished by its elevated level of resting metabolic activity, and its connectivity is implicated in a broad spectrum of cognitive, emotional, and social functions [43, 44]. Notably, REM connectome differences in the DMN (within and between the network), regardless of individual or group level, predicted the REM-sleep loss effect. These findings are consistent with previous research that has linked connectivity in the DMN vulnerable to extreme alterations in lack of sleep [45]. However, the late-night sleep deprivation results in the most pronounced reduction in connections within the DMN. This indicates that both late-night and early-night sleep deprivation result in insufficient REM sleep, but late-night sleep deprivation appears to exert a more detrimental influence compared to early-night sleep deprivation. It is therefore noteworthy that the absence of REM sleep, which exacerbate the incidence and pathogenesis of psychiatric disorders [3, 46], and that the late-night may provide a better time window to improve and treat psychiatric disorders.

One limitation of our study is that we have only investigated the brain activity related to REM sleep without linking to the behavior changes, such as memory and cognitive, which could be explored in more depth in the future. Moreover, we did not input the NREM sleep deprivation into analysis, since there are already a number of studies have conducted on the NREM sleep, which is more involved in semantic memory consolidation and maintaining sleep stability [39–41].

In summary, insufficient REM sleep disrupts the dynamic reorganization of resting-state functional brain networks, primarily affecting the DMN Network. Additionally, the edges of the thalamus contribute significantly to these disruptions in connectivity. The present study contributes to our understanding of the role of the REM sleep phase in maintaining or modifying brain variability to some extent, and highlights possible neural mechanisms supporting connectivity within large brain networks when sleep is inadequate. Future investigations should consider integrating real-time measures of REM sleep with fMRI to ascertain the dynamic association between REM related connectivity with REM sleep. And investigate into the effects of total REM sleep deprivation might provide more valuable context for the interpretation of brain-behavior predictive modeling.

### Supplementary information

supplement table

## Data Availability

The data that support the findings of this study are available from the corresponding author, Yan Sun and Jie Shi, upon reasonable request.
