# Supplementary material for: The impact of REM sleep loss on human brain connectivity
Source: Transl Psychiatry. 2024 Jul 2;14:270. doi: 10.1038/s41398-024-02985-x (PMC11219886; doi:10.1038/s41398-024-02985-x)
Supplement: Supplementary file 1 — supplement table [file 41398_2024_2985_MOESM1_ESM.docx]

**Supplement**

**Table S1 The ROI information used in the present study**

| **ROI** | **FSL_Label** | **MNI-X** | **MNI-Y** | **MNI-Z** | **Color** | **Brain Networks** |
| --- | --- | --- | --- | --- | --- | --- |
| 1 | precuneus | -8 | -54 | 57 | Cyan | Sensory/somatomotor |
| 2 | cingulate | -14 | -21 | 39 | Cyan | Sensory/somatomotor |
| 3 | cingulate | -1 | -18 | 46 | Cyan | Sensory/somatomotor |
| 4 | cingulate | 8 | -6 | 45 | Cyan | Sensory/somatomotor |
| 5 | med_precentral_gyrus | -8 | -24 | 63 | Cyan | Sensory/somatomotor |
| 6 | postcentral_gyrus | -8 | -36 | 69 | Cyan | Sensory/somatomotor |
| 7 | postcentral_gyrus | 11 | -36 | 72 | Cyan | Sensory/somatomotor |
| 8 | postcentral_gyrus | -52 | -25 | 41 | Cyan | Sensory/somatomotor |
| 9 | precentral_gyrus | 26 | -21 | 69 | Cyan | Sensory/somatomotor |
| 10 | postcentral_gyrus | 8 | -48 | 69 | Cyan | Sensory/somatomotor |
| 11 | postcentral_gyrus | -23 | -33 | 69 | Cyan | Sensory/somatomotor |
| 12 | precentral_gyrus | -39 | -22 | 52 | Cyan | Sensory/somatomotor |
| 13 | SPL | 26 | -42 | 57 | Cyan | Sensory/somatomotor |
| 14 | postcentral_gyrus/IPL | 47 | -24 | 42 | Cyan | Sensory/somatomotor |
| 15 | postcentral_gyrus | -38 | -30 | 66 | Cyan | Sensory/somatomotor |
| 16 | precentral_gyrus | 18 | -32 | 58 | Cyan | Sensory/somatomotor |
| 17 | precentral_gyrus | 41 | -12 | 57 | Cyan | Sensory/somatomotor |
| 18 | SPL/IPL | -29 | -45 | 57 | Cyan | Sensory/somatomotor |
| 19 | precentral_gyrus | 8 | -21 | 72 | Cyan | Sensory/somatomotor |
| 20 | SPL | 20 | -45 | 66 | Cyan | Sensory/somatomotor |
| 21 | postcentral_gyrus/SMG_border | -44 | -34 | 44 | Cyan | Sensory/somatomotor |
| 22 | postcentral_gyrus | -21 | -34 | 58 | Cyan | Sensory/somatomotor |
| 23 | precentral_gyrus | -14 | -21 | 72 | Cyan | Sensory/somatomotor |
| 24 | postcentral_gyrus | 39 | -24 | 54 | Cyan | Sensory/somatomotor |
| 25 | precentral_gyrus | -38 | -18 | 66 | Cyan | Sensory/somatomotor |
| 26 | postcentral_gyrus | -17 | -48 | 69 | Cyan | Sensory/somatomotor |
| 27 | med_precentral_gyrus | 1 | -31 | 58 | Cyan | Sensory/somatomotor |
| 28 | med_precentral_gyrus | 2 | -21 | 57 | Cyan | Sensory/somatomotor |
| 29 | central_sulcus | 35 | -21 | 45 | Cyan | Sensory/somatomotor |
| 30 | precentral_gyrus | -48 | -14 | 34 | Cyan | Sensory/somatomotor |
| 31 | insula | 34 | -13 | 16 | Cyan | Sensory/somatomotor |
| 32 | precentral_gyrus | 48 | -10 | 34 | Cyan | Sensory/somatomotor |
| 33 | postcentral_gyrus | -51 | -13 | 24 | Cyan | Sensory/somatomotor |
| 34 | postcentral_gyrus | 62 | -12 | 27 | Cyan | Sensory/somatomotor |
| 35 | SMA | -4 | -2 | 53 | Purple | Cingulo-opercular Task Control |
| 36 | SMG | 51 | -31 | 34 | Purple | Cingulo-opercular Task Control |
| 37 | SFG | 17 | -12 | 63 | Purple | Cingulo-opercular Task Control |
| 38 | SFG | -17 | -9 | 69 | Purple | Cingulo-opercular Task Control |
| 39 | cingulate/SMA | -11 | -6 | 42 | Purple | Cingulo-opercular Task Control |
| 40 | insula | 35 | -3 | 0 | Purple | Cingulo-opercular Task Control |
| 41 | SFG | 11 | -6 | 69 | Purple | Cingulo-opercular Task Control |
| 42 | SMA | 5 | 3 | 51 | Purple | Cingulo-opercular Task Control |
| 43 | central_operculum/insula | -43 | -3 | 10 | Purple | Cingulo-opercular Task Control |
| 44 | central_operculum/insula | 47 | 4 | 3 | Purple | Cingulo-opercular Task Control |
| 45 | insula | -33 | 0 | 6 | Purple | Cingulo-opercular Task Control |
| 46 | temporal_pole_sup | -49 | 5 | 0 | Purple | Cingulo-opercular Task Control |
| 47 | ACC | -6 | 13 | 36 | Purple | Cingulo-opercular Task Control |
| 48 | insula | 34 | 6 | 5 | Purple | Cingulo-opercular Task Control |
| 49 | heschls_gyrus | 30 | -29 | 14 | Pink | Auditory |
| 50 | STG | 62 | -36 | 21 | Pink | Auditory |
| 51 | STG | 55 | -19 | 10 | Pink | Auditory |
| 52 | planum_temporale/STG? | -37 | -35 | 16 | Pink | Auditory |
| 53 | planum_temporale/STG | -58 | -27 | 13 | Pink | Auditory |
| 54 | STG/planum_temporale | -47 | -28 | 5 | Pink | Auditory |
| 55 | parietal_operculum/insula | 41 | -26 | 21 | Pink | Auditory |
| 56 | parietal_operculum/SMG | -48 | -36 | 24 | Pink | Auditory |
| 57 | postcentral_gyrus/central_operculum | -51 | -24 | 22 | Pink | Auditory |
| 58 | parietal_operculum | -53 | -12 | 12 | Pink | Auditory |
| 59 | parietal_operculum | 53 | -9 | 16 | Pink | Auditory |
| 60 | postcentral_gyrus | 56 | -21 | 30 | Pink | Auditory |
| 61 | pos_insula | -29 | -29 | 12 | Pink | Auditory |
| 62 | LOC | -39 | -75 | 22 | Red | Default mode |
| 63 | mFC/paracingulate | 5 | 60 | 3 | Red | Default mode |
| 64 | mFC/paracingulate | 8 | 42 | -9 | Red | Default mode |
| 65 | PCC/parahipp_gyrus | -12 | -41 | 1 | Red | Default mode |
| 66 | frontal_pole_inf/SFG | -17 | 57 | -3 | Red | Default mode |
| 67 | LOC/MTG | -44 | -61 | 18 | Red | Default mode |
| 68 | LOC | 41 | -73 | 26 | Red | Default mode |
| 69 | temporal_pole | -41 | 9 | -30 | Red | Default mode |
| 70 | temporal_pole | 44 | 12 | -24 | Red | Default mode |
| 71 | MTG | -65 | -24 | -15 | Red | Default mode |
| 72 | LOC/AG | -43 | -65 | 31 | Red | Default mode |
| 73 | LOC/parietal_border | -38 | -75 | 39 | Red | Default mode |
| 74 | precuneus/PCC | -7 | -56 | 25 | Red | Default mode |
|  |  |  |  |  |  |  |
| 75 | precuneus | 5 | -60 | 33 | Red | Default mode |
| 76 | precuneus/PCC | -11 | -57 | 14 | Red | Default mode |
| 77 | PCC | -3 | -50 | 12 | Red | Default mode |
| 78 | PCC | 7 | -50 | 29 | Red | Default mode |
| 79 | precuneus | 14 | -64 | 24 | Red | Default mode |
| 80 | cingulate/PCC | -3 | -39 | 42 | Red | Default mode |
| 81 | precuneus/PCC | 10 | -55 | 16 | Red | Default mode |
| 82 | LOC/AG | 49 | -61 | 34 | Red | Default mode |
| 83 | SFG | 21 | 27 | 50 | Red | Default mode |
| 84 | SFG | -11 | 33 | 54 | Red | Default mode |
| 85 | SFG | -17 | 23 | 54 | Red | Default mode |
| 86 | MFG | -35 | 15 | 51 | Red | Default mode |
| 87 | SFG | 20 | 33 | 42 | Red | Default mode |
| 88 | frontal_pole_sup/SFG | 11 | 48 | 42 | Red | Default mode |
| 89 | frontal_pole_sup/SFG | -11 | 48 | 42 | Red | Default mode |
| 90 | frontal_pole_sup/SFG | -20 | 39 | 42 | Red | Default mode |
| 91 | medial_frontal_gyrus | 5 | 48 | 21 | Red | Default mode |
| 92 | frontal_pole_sup/SFG | 5 | 57 | 27 | Red | Default mode |
| 93 | mFG/cingulate | -7 | 45 | 4 | Red | Default mode |
| 94 | mFC/paracingulate | 8 | 48 | 9 | Red | Default mode |
| 95 | mFC/paracingulate | -3 | 39 | -4 | Red | Default mode |
| 96 | mFC/paracingulate | 7 | 37 | 0 | Red | Default mode |
| 97 | mFG/cingulate | -11 | 39 | 12 | Red | Default mode |
| 98 | mFC/paracingulate | -3 | 32 | 39 | Red | Default mode |
| 99 | cingulate | -3 | 36 | 20 | Red | Default mode |
| 100 | frontal_pole/SFG | -20 | 57 | 24 | Red | Default mode |
| 101 | mFC/paracingulate | -8 | 42 | 27 | Red | Default mode |
| 102 | MTG/ITG | 62 | -15 | -15 | Red | Default mode |
| 103 | MTG | -53 | -15 | -9 | Red | Default mode |
| 104 | MTG | -55 | -31 | -4 | Red | Default mode |
| 105 | MTG | 62 | -33 | -6 | Red | Default mode |
| 106 | MTG | -65 | -42 | -6 | Red | Default mode |
| 107 | SFG | 11 | 24 | 60 | Red | Default mode |
| 108 | cingulate | 11 | 30 | 24 | Red | Default mode |
| 109 | MTG | 50 | -6 | -12 | Red | Default mode |
| 110 | parahipp_gyrus | -25 | -41 | -8 | Red | Default mode |
| 111 | parahipp_gyrus | 26 | -39 | -11 | Red | Default mode |
| 112 | fusiform | -32 | -39 | -15 | Red | Default mode |
| 113 | cerebellum | 28 | -76 | -31 | Red | Default mode |
| 114 | temporal_pole/MTG | 50 | 3 | -24 | Red | Default mode |
| 115 | MTG | -50 | 0 | -24 | Red | Default mode |
| 116 | AG | 44 | -52 | 28 | Red | Default mode |
| 117 | MTG | -47 | -43 | 0 | Red | Default mode |
| 118 | OFC/IFG | -44 | 27 | -9 | Red | Default mode |
| 119 | SFG/SMA | -11 | 6 | 66 | Teal | Ventral attention |
| 120 | frontal_pole_inf/IFG | 47 | 30 | -6 | Red | Default mode |
| 121 | lingual_gyrus | 17 | -48 | -9 | Blue | Visual |
| 122 | LOC | 38 | -73 | 13 | Blue | Visual |
| 123 | occip_cortex/intracalcarine | 8 | -72 | 9 | Blue | Visual |
| 124 | occip_cortex/intracalcarine | -8 | -80 | 5 | Blue | Visual |
| 125 | LOC | -27 | -79 | 16 | Blue | Visual |
| 126 | lingual_gyrus | 19 | -66 | 1 | Blue | Visual |
| 127 | occip_pole/middle_occip_gyrus | -23 | -90 | 15 | Blue | Visual |
| 128 | fusiform | 26 | -60 | -9 | Blue | Visual |
| 129 | lingual_gyrus | -14 | -72 | -9 | Blue | Visual |
| 130 | occip_cortex/intracalcarine | -17 | -68 | 3 | Blue | Visual |
| 131 | inferior_occip_cortex | 41 | -78 | -12 | Blue | Visual |
| 132 | inferior_occip_cortex | -44 | -75 | -12 | Blue | Visual |
| 133 | occip_pole_sup | -14 | -90 | 27 | Blue | Visual |
| 134 | occip_cortex_sup | 14 | -87 | 33 | Blue | Visual |
| 135 | LOC | 27 | -77 | 23 | Blue | Visual |
| 136 | occip_cortex/fusiform | 19 | -85 | -4 | Blue | Visual |
| 137 | occip_cortex/cuneus | 14 | -77 | 28 | Blue | Visual |
| 138 | lingual_gyrus | -15 | -53 | -2 | Blue | Visual |
| 139 | LOC/occip_cortex | 40 | -66 | -8 | Blue | Visual |
| 140 | LOC | 23 | -87 | 21 | Blue | Visual |
| 141 | occip_cortex/cuneus | 5 | -72 | 21 | Blue | Visual |
| 142 | LOC | -40 | -73 | -2 | Blue | Visual |
| 143 | fusiform | 25 | -79 | -16 | Blue | Visual |
| 144 | occip_cortex/cuneus | -16 | -77 | 30 | Blue | Visual |
| 145 | occip_cortex/cuneus | -3 | -81 | 18 | Blue | Visual |
| 146 | LOC/inf_occip_gyrus | -38 | -87 | -9 | Blue | Visual |
| 147 | LOC | 35 | -84 | 11 | Blue | Visual |
| 148 | occip_cortex/intracalcarine | 6 | -81 | 4 | Blue | Visual |
| 149 | occip_pole | -25 | -89 | 0 | Blue | Visual |
| 150 | fusiform | -31 | -78 | -15 | Blue | Visual |
| 151 | LOC | 35 | -81 | 0 | Blue | Visual |
| 152 | precentral_gyrus | -43 | -2 | 45 | Yellow | Fronto-parietal Task Control |
| 153 | MFG | 45 | 19 | 30 | Yellow | Fronto-parietal Task Control |
| 154 | IFG | -45 | 7 | 24 | Yellow | Fronto-parietal Task Control |
| 155 | SMG | -51 | -50 | 39 | Yellow | Fronto-parietal Task Control |
| 156 | SFG | -23 | 6 | 63 | Yellow | Fronto-parietal Task Control |
| 157 | ITG | 56 | -54 | -12 | Yellow | Fronto-parietal Task Control |
| 158 | frontal_pole_inf/MFG | 23 | 39 | -9 | Yellow | Fronto-parietal Task Control |
| 159 | frontal_pole_inf/MFG | 32 | 48 | -6 | Yellow | Fronto-parietal Task Control |
| 160 | precentral_gyrus | 44 | 5 | 35 | Yellow | Fronto-parietal Task Control |
| 161 | precentral_gyrus/MFG | -40 | 2 | 33 | Yellow | Fronto-parietal Task Control |
| 162 | MFG | -41 | 33 | 24 | Yellow | Fronto-parietal Task Control |
| 163 | MFG/frontal_pole | 36 | 37 | 20 | Yellow | Fronto-parietal Task Control |
| 164 | SMG | 46 | -45 | 44 | Yellow | Fronto-parietal Task Control |
| 165 | SPL | -28 | -59 | 44 | Yellow | Fronto-parietal Task Control |
| 166 | AG | 41 | -55 | 45 | Yellow | Fronto-parietal Task Control |
| 167 | MFG_sup | 29 | 9 | 57 | Yellow | Fronto-parietal Task Control |
| 168 | LOC/parietal_border | 35 | -66 | 38 | Yellow | Fronto-parietal Task Control |
| 169 | AG | -41 | -56 | 41 | Yellow | Fronto-parietal Task Control |
| 170 | MFG | 37 | 13 | 42 | Yellow | Fronto-parietal Task Control |
| 171 | frontal_pole/MFG | -33 | 49 | 9 | Yellow | Fronto-parietal Task Control |
| 172 | frontal_pole/IFG | -40 | 40 | 2 | Yellow | Fronto-parietal Task Control |
| 173 | SPL/IPL | 31 | -55 | 42 | Yellow | Fronto-parietal Task Control |
| 174 | frontal_pole | 41 | 43 | 4 | Yellow | Fronto-parietal Task Control |
| 175 | MFG | -41 | 20 | 31 | Yellow | Fronto-parietal Task Control |
| 176 | mFC/paracingulate | -4 | 21 | 46 | Yellow | Fronto-parietal Task Control |
| 177 | precuneus | 9 | -41 | 48 | Black | Salience |
| 178 | SMG | 52 | -47 | 36 | Black | Salience |
| 179 | precentral_gyrus | 39 | -5 | 48 | Black | Salience |
| 180 | MFG | 29 | 27 | 30 | Black | Salience |
| 181 | IFG | 45 | 17 | 14 | Black | Salience |
| 182 | insula | -34 | 16 | 3 | Black | Salience |
| 183 | insula | 34 | 17 | 7 | Black | Salience |
| 184 | insula | 35 | 27 | 3 | Black | Salience |
| 185 | insula | 32 | 12 | -3 | Black | Salience |
| 186 | ACC | -11 | 21 | 27 | Black | Salience |
| 187 | mFC/paracingulate | -2 | 10 | 45 | Black | Salience |
| 188 | frontal_pole_sup/SFG | -27 | 46 | 25 | Black | Salience |
| 189 | ACC | -1 | 25 | 30 | Black | Salience |
| 190 | ACC | 4 | 18 | 39 | Black | Salience |
| 191 | ACC | 9 | 17 | 30 | Black | Salience |
| 192 | frontal_pole_sup/SFG | 29 | 49 | 20 | Black | Salience |
| 193 | frontal_pole_sup/SFG | 24 | 43 | 31 | Black | Salience |
| 194 | frontal_pole_sup/MFG | -38 | 45 | 21 | Black | Salience |
| 195 | thalamus | 6 | -26 | 1 | Brown | Subcortical |
| 196 | thalamus | -2 | -16 | 13 | Brown | Subcortical |
| 197 | thalamus | -10 | -21 | 8 | Brown | Subcortical |
| 198 | thalamus | 11 | -20 | 9 | Brown | Subcortical |
| 199 | brainstem/SC | -5 | -30 | -3 | Brown | Subcortical |
| 200 | putamen | -21 | 4 | -2 | Brown | Subcortical |
| 201 | putamen/white_matter | -15 | 0 | 10 | Brown | Subcortical |
| 202 | putamen | 29 | -17 | 4 | Brown | Subcortical |
| 203 | putamen | 22 | 6 | 5 | Brown | Subcortical |
| 204 | putamen | 27 | -3 | 7 | Brown | Subcortical |
| 205 | putamen | -30 | -14 | 1 | Brown | Subcortical |
| 206 | putamen/white_matter | 14 | 1 | 10 | Brown | Subcortical |
| 207 | thalamus | 8 | -7 | 8 | Brown | Subcortical |
| 208 | SMG/STG/TPJ | 51 | -45 | 22 | Teal | Ventral attention |
| 209 | MTG | -54 | -51 | 8 | Teal | Ventral attention |
| 210 | STG | -53 | -41 | 12 | Teal | Ventral attention |
| 211 | STG | 49 | -35 | 9 | Teal | Ventral attention |
| 212 | MTG | 49 | -31 | -2 | Teal | Ventral attention |
| 213 | MTG/STG | 53 | -48 | 12 | Teal | Ventral attention |
| 214 | IFG | 50 | 27 | 6 | Teal | Ventral attention |
| 215 | IFG | -47 | 21 | 2 | Teal | Ventral attention |
| 216 | SPL | 8 | -63 | 57 | Green | Dorsal attention |
| 217 | MTG/LOC | -50 | -63 | 3 | Green | Dorsal attention |
| 218 | postcentral_gyrus/SMG_border | 44 | -33 | 48 | Cyan | Sensory/somatomotor |
| 219 | SPL | 20 | -66 | 45 | Green | Dorsal attention |
| 220 | MTG/LOC | 44 | -60 | 4 | Green | Dorsal attention |
| 221 | SPL | 23 | -60 | 57 | Green | Dorsal attention |
| 222 | SPL | -32 | -48 | 44 | Green | Dorsal attention |
| 223 | LOC/parietal_border | -26 | -71 | 33 | Green | Dorsal attention |
| 224 | MFG | -32 | -5 | 53 | Green | Dorsal attention |
| 225 | ITG/fusiform | -40 | -60 | -10 | Green | Dorsal attention |
| 226 | SPL | -17 | -60 | 60 | Green | Dorsal attention |
| 227 | precentral_gyrus | 26 | -9 | 54 | Green | Dorsal attention |

**Table S2a. The brain network edges in the REM connectome**

|  | SMN | CON | AUD | DMN | VIS | FPN | SAN | SUN | VAN | DAN |
| --- | --- | --- | --- | --- | --- | --- | --- | --- | --- | --- |
| SMN | 0 | 1 | 4 | 3 | 0 | 0 | 1 | 4 | 3 | 0 |
| CON | 1 | 10 | 5 | 0 | 0 | 0 | 2 | 1 | 0 | 3 |
| AUD | 4 | 5 | 0 | 0 | 2 | 0 | 2 | 0 | 0 | 2 |
| DMN | 3 | 0 | 0 | 16 | 15 | 0 | 0 | 7 | 0 | 1 |
| VIS | 0 | 0 | 2 | 15 | 4 | 6 | 4 | 11 | 1 | 0 |
| FPN | 0 | 0 | 0 | 0 | 6 | 4 | 6 | 3 | 1 | 0 |
| SAN | 1 | 2 | 2 | 0 | 4 | 6 | 4 | 1 | 0 | 1 |
| SUN | 4 | 1 | 0 | 7 | 11 | 3 | 1 | 0 | 1 | 0 |
| VAN | 3 | 0 | 0 | 0 | 1 | 1 | 0 | 1 | 0 | 0 |
| DAN | 0 | 3 | 2 | 1 | 0 | 0 | 1 | 0 | 0 | 2 |

**Table S2b. The brain network correlation in the REM connectome**

|  | SMN | CON | AUD | DMN | VIS | FPN | SAN | SUN | VAN | DAN |
| --- | --- | --- | --- | --- | --- | --- | --- | --- | --- | --- |
| SMN | 0.00 | 0.32 | 0.40 | 0.38 | 0.00 | 0.00 | 0.28 | 0.30 | 0.37 | 0.00 |
| CON | 0.32 | 0.37 | 0.45 | 0.00 | 0.00 | 0.00 | 0.37 | 0.28 | 0.00 | 0.36 |
| AUD | 0.40 | 0.45 | 0.00 | 0.00 | 0.33 | 0.00 | 0.33 | 0.00 | 0.00 | 0.36 |
| DMN | 0.38 | 0.00 | 0.00 | 0.47 | 0.49 | 0.00 | 0.00 | 0.42 | 0.00 | 0.26 |
| VIS | 0.00 | 0.00 | 0.33 | 0.49 | 0.29 | 0.35 | 0.31 | 0.33 | 0.25 | 0.00 |
| FPN | 0.00 | 0.00 | 0.00 | 0.00 | 0.35 | 0.34 | 0.44 | 0.32 | 0.29 | 0.00 |
| SAN | 0.28 | 0.37 | 0.33 | 0.00 | 0.31 | 0.44 | 0.37 | 0.28 | 0.00 | 0.26 |
| SUN | 0.30 | 0.28 | 0.00 | 0.42 | 0.33 | 0.32 | 0.28 | 0.00 | 0.27 | 0.00 |
| VAN | 0.37 | 0.00 | 0.00 | 0.00 | 0.25 | 0.29 | 0.00 | 0.27 | 0.00 | 0.00 |
| DAN | 0.00 | 0.36 | 0.36 | 0.26 | 0.00 | 0.00 | 0.26 | 0.00 | 0.00 | 0.27 |

**Table S3. The large-scale network importance and contribution in the REM connectome**

| Large-scale Networks | Edges | Correlation |
| --- | --- | --- |
| SMN | 16 | 0.55 |
| CON | 22 | 0.48 |
| AUD | 15 | 0.53 |
| DMN | 42 | 0.62 |
| VIS | 43 | 0.53 |
| FPN | 20 | 0.52 |
| SAN | 21 | 0.52 |
| SUN | 28 | 0.44 |
| VAN | 6 | 0.50 |
| DAN | 9 | 0.42 |

**Table S4. The ROI importance and contribution in the REM connectome**

| ROI-number | ROI-label | Large-scale network | ROI-degree | ROI-corr |
| --- | --- | --- | --- | --- |
| 1 | precuneus | Sensory somatomotor | 1 | 0.29 |
| 2 | cingulate | Sensory somatomotor | 0 | 0.00 |
| 3 | cingulate | Sensory somatomotor | 0 | 0.00 |
| 4 | cingulate | Sensory somatomotor | 0 | 0.00 |
| 5 | med_precentral_gyrus | Sensory somatomotor | 0 | 0.00 |
| 6 | postcentral_gyrus | Sensory somatomotor | 0 | 0.00 |
| 7 | postcentral_gyrus | Sensory somatomotor | 1 | 0.29 |
| 8 | postcentral_gyrus | Sensory somatomotor | 0 | 0.00 |
| 9 | precentral_gyrus | Sensory somatomotor | 3 | 0.35 |
| 10 | postcentral_gyrus | Sensory somatomotor | 0 | 0.00 |
| 11 | postcentral_gyrus | Sensory somatomotor | 1 | 0.26 |
| 12 | precentral_gyrus | Sensory somatomotor | 0 | 0.00 |
| 13 | SPL | Sensory somatomotor | 2 | 0.36 |
| 14 | postcentral_gyrus/IPL | Sensory somatomotor | 0 | 0.00 |
| 15 | postcentral_gyrus | Sensory somatomotor | 0 | 0.00 |
| 16 | precentral_gyrus | Sensory somatomotor | 0 | 0.00 |
| 17 | precentral_gyrus | Sensory somatomotor | 1 | 0.26 |
| 18 | SPL/IPL | Sensory somatomotor | 0 | 0.00 |
| 19 | precentral_gyrus | Sensory somatomotor | 0 | 0.00 |
| 20 | SPL | Sensory somatomotor | 0 | 0.00 |
| 21 | postcentral_gyrus/SMG_border | Sensory somatomotor | 0 | 0.00 |
| 22 | postcentral_gyrus | Sensory somatomotor | 0 | 0.00 |
| 23 | precentral_gyrus | Sensory somatomotor | 0 | 0.00 |
| 24 | postcentral_gyrus | Sensory somatomotor | 0 | 0.00 |
| 25 | precentral_gyrus | Sensory somatomotor | 0 | 0.00 |
| 26 | postcentral_gyrus | Sensory somatomotor | 4 | 0.40 |
| 27 | med_precentral_gyrus | Sensory somatomotor | 0 | 0.00 |
| 28 | med_precentral_gyrus | Sensory somatomotor | 0 | 0.00 |
| 29 | central_sulcus | Sensory somatomotor | 3 | 0.32 |
| 30 | precentral_gyrus | Sensory somatomotor | 0 | 0.00 |
| 31 | insula | Sensory somatomotor | 0 | 0.00 |
| 32 | precentral_gyrus | Sensory somatomotor | 0 | 0.00 |
| 33 | postcentral_gyrus | Sensory somatomotor | 0 | 0.00 |
| 34 | postcentral_gyrus | Sensory somatomotor | 0 | 0.00 |
| 35 | SMA | Cingulo-opercular Task Control | 0 | 0.00 |
| 36 | SMG | Cingulo-opercular Task Control | 6 | 0.39 |
| 37 | SFG | Cingulo-opercular Task Control | 0 | 0.00 |
| 38 | SFG | Cingulo-opercular Task Control | 3 | 0.34 |
| 39 | cingulate/SMA | Cingulo-opercular Task Control | 4 | 0.40 |
| 40 | insula | Cingulo-opercular Task Control | 0 | 0.00 |
| 41 | SFG | Cingulo-opercular Task Control | 2 | 0.33 |
| 42 | SMA | Cingulo-opercular Task Control | 0 | 0.00 |
| 43 | central_operculum/insula | Cingulo-opercular Task Control | 6 | 0.40 |
| 44 | central_operculum/insula | Cingulo-opercular Task Control | 0 | 0.00 |
| 45 | insula | Cingulo-opercular Task Control | 0 | 0.00 |
| 46 | temporal_pole_sup | Cingulo-opercular Task Control | 1 | 0.25 |
| 47 | ACC | Cingulo-opercular Task Control | 0 | 0.00 |
| 48 | insula | Cingulo-opercular Task Control | 0 | 0.00 |
| 49 | heschls_gyrus | Auditory | 0 | 0.00 |
| 50 | STG | Auditory | 8 | 0.43 |
| 51 | STG | Auditory | 1 | 0.27 |
| 52 | planum_temporale/STG? | Auditory | 1 | 0.26 |
| 53 | planum_temporale/STG | Auditory | 0 | 0.00 |
| 54 | STG/planum_temporale | Auditory | 2 | 0.31 |
| 55 | parietal_operculum/insula | Auditory | 0 | 0.00 |
| 56 | parietal_operculum/SMG | Auditory | 1 | 0.28 |
| 57 | postcentral_gyrus/central_operculum | Auditory | 0 | 0.00 |
| 58 | parietal_operculum | Auditory | 1 | 0.29 |
| 59 | parietal_operculum | Auditory | 0 | 0.00 |
| 60 | postcentral_gyrus | Auditory | 1 | 0.29 |
| 61 | pos_insula | Auditory | 0 | 0.00 |
| 62 | LOC | Default mode | 0 | 0.00 |
| 63 | mFC/paracingulate | Default mode | 5 | 0.38 |
| 64 | mFC/paracingulate | Default mode | 2 | 0.34 |
| 65 | PCC/parahipp_gyrus | Default mode | 0 | 0.00 |
| 66 | frontal_pole_inf/SFG | Default mode | 0 | 0.00 |
| 67 | LOC/MTG | Default mode | 0 | 0.00 |
| 68 | LOC | Default mode | 0 | 0.00 |
| 69 | temporal_pole | Default mode | 0 | 0.00 |
| 70 | temporal_pole | Default mode | 0 | 0.00 |
| 71 | MTG | Default mode | 0 | 0.00 |
| 72 | LOC/AG | Default mode | 0 | 0.00 |
| 73 | LOC/parietal_border | Default mode | 0 | 0.00 |
| 74 | precuneus/PCC | Default mode | 5 | 0.46 |
| 75 | precuneus | Default mode | 1 | 0.26 |
| 76 | precuneus/PCC | Default mode | 0 | 0.00 |
| 77 | PCC | Default mode | 2 | 0.35 |
| 78 | PCC | Default mode | 0 | 0.00 |
| 79 | precuneus | Default mode | 0 | 0.00 |
| 80 | cingulate/PCC | Default mode | 0 | 0.00 |
| 81 | precuneus/PCC | Default mode | 0 | 0.00 |
| 82 | LOC/AG | Default mode | 0 | 0.00 |
| 83 | SFG | Default mode | 0 | 0.00 |
| 84 | SFG | Default mode | 0 | 0.00 |
| 85 | SFG | Default mode | 0 | 0.00 |
| 86 | MFG | Default mode | 0 | 0.00 |
| 87 | SFG | Default mode | 0 | 0.00 |
| 88 | frontal_pole_sup/SFG | Default mode | 3 | 0.33 |
| 89 | frontal_pole_sup/SFG | Default mode | 6 | 0.40 |
| 90 | frontal_pole_sup/SFG | Default mode | 0 | 0.00 |
| 91 | medial_frontal_gyrus | Default mode | 0 | 0.00 |
| 92 | frontal_pole_sup/SFG | Default mode | 6 | 0.41 |
| 93 | mFG/cingulate | Default mode | 0 | 0.00 |
| 94 | mFC/paracingulate | Default mode | 0 | 0.00 |
| 95 | mFC/paracingulate | Default mode | 1 | 0.26 |
| 96 | mFC/paracingulate | Default mode | 2 | 0.35 |
| 97 | mFG/cingulate | Default mode | 0 | 0.00 |
| 98 | mFC/paracingulate | Default mode | 0 | 0.00 |
| 99 | cingulate | Default mode | 0 | 0.00 |
| 100 | frontal_pole/SFG | Default mode | 0 | 0.00 |
| 101 | mFC/paracingulate | Default mode | 0 | 0.00 |
| 102 | MTG/ITG | Default mode | 3 | 0.33 |
| 103 | MTG | Default mode | 0 | 0.00 |
| 104 | MTG | Default mode | 0 | 0.00 |
| 105 | MTG | Default mode | 0 | 0.00 |
| 106 | MTG | Default mode | 0 | 0.00 |
| 107 | SFG | Default mode | 0 | 0.00 |
| 108 | cingulate | Default mode | 1 | 0.25 |
| 109 | MTG | Default mode | 0 | 0.00 |
| 110 | parahipp_gyrus | Default mode | 0 | 0.00 |
| 111 | parahipp_gyrus | Default mode | 0 | 0.00 |
| 112 | fusiform | Default mode | 1 | 0.27 |
| 113 | cerebellum | Default mode | 1 | 0.27 |
| 114 | temporal_pole/MTG | Default mode | 0 | 0.00 |
| 115 | MTG | Default mode | 0 | 0.00 |
| 116 | AG | Default mode | 0 | 0.00 |
| 117 | MTG | Default mode | 0 | 0.00 |
| 118 | OFC/IFG | Default mode | 0 | 0.00 |
| 119 | SFG/SMA | Ventral attention | 0 | 0.00 |
| 120 | frontal_pole_inf/IFG | Default mode | 3 | 0.32 |
| 121 | lingual_gyrus | Visual | 0 | 0.00 |
| 122 | LOC | Visual | 0 | 0.00 |
| 123 | occip_cortex/intracalcarine | Visual | 7 | 0.46 |
| 124 | occip_cortex/intracalcarine | Visual | 9 | 0.42 |
| 125 | LOC | Visual | 0 | 0.00 |
| 126 | lingual_gyrus | Visual | 2 | 0.32 |
| 127 | occip_pole/middle_occip_gyrus | Visual | 0 | 0.00 |
| 128 | fusiform | Visual | 0 | 0.00 |
| 129 | lingual_gyrus | Visual | 1 | 0.26 |
| 130 | occip_cortex/intracalcarine | Visual | 2 | 0.33 |
| 131 | inferior_occip_cortex | Visual | 0 | 0.00 |
| 132 | inferior_occip_cortex | Visual | 0 | 0.00 |
| 133 | occip_pole_sup | Visual | 1 | 0.25 |
| 134 | occip_cortex_sup | Visual | 0 | 0.00 |
| 135 | LOC | Visual | 0 | 0.00 |
| 136 | occip_cortex/fusiform | Visual | 3 | 0.35 |
| 137 | occip_cortex/cuneus | Visual | 0 | 0.00 |
| 138 | lingual_gyrus | Visual | 8 | 0.43 |
| 139 | LOC/occip_cortex | Visual | 0 | 0.00 |
| 140 | LOC | Visual | 1 | 0.25 |
| 141 | occip_cortex/cuneus | Visual | 0 | 0.00 |
| 142 | LOC | Visual | 0 | 0.00 |
| 143 | fusiform | Visual | 0 | 0.00 |
| 144 | occip_cortex/cuneus | Visual | 0 | 0.00 |
| 145 | occip_cortex/cuneus | Visual | 0 | 0.00 |
| 146 | LOC/inf_occip_gyrus | Visual | 0 | 0.00 |
| 147 | LOC | Visual | 0 | 0.00 |
| 148 | occip_cortex/intracalcarine | Visual | 8 | 0.45 |
| 149 | occip_pole | Visual | 0 | 0.00 |
| 150 | fusiform | Visual | 0 | 0.00 |
| 151 | LOC | Visual | 1 | 0.27 |
| 152 | precentral_gyrus | Fronto-parietal Task Control | 0 | 0.00 |
| 153 | MFG | Fronto-parietal Task Control | 2 | 0.31 |
| 154 | IFG | Fronto-parietal Task Control | 0 | 0.00 |
| 155 | SMG | Fronto-parietal Task Control | 1 | 0.32 |
| 156 | SFG | Fronto-parietal Task Control | 6 | 0.38 |
| 157 | ITG | Fronto-parietal Task Control | 0 | 0.00 |
| 158 | frontal_pole_inf/MFG | Fronto-parietal Task Control | 3 | 0.40 |
| 159 | frontal_pole_inf/MFG | Fronto-parietal Task Control | 0 | 0.00 |
| 160 | precentral_gyrus | Fronto-parietal Task Control | 0 | 0.00 |
| 161 | precentral_gyrus/MFG | Fronto-parietal Task Control | 0 | 0.00 |
| 162 | MFG | Fronto-parietal Task Control | 0 | 0.00 |
| 163 | MFG/frontal_pole | Fronto-parietal Task Control | 0 | 0.00 |
| 164 | SMG | Fronto-parietal Task Control | 1 | 0.30 |
| 165 | SPL | Fronto-parietal Task Control | 0 | 0.00 |
| 166 | AG | Fronto-parietal Task Control | 1 | 0.28 |
| 167 | MFG_sup | Fronto-parietal Task Control | 0 | 0.00 |
| 168 | LOC/parietal_border | Fronto-parietal Task Control | 0 | 0.00 |
| 169 | AG | Fronto-parietal Task Control | 0 | 0.00 |
| 170 | MFG | Fronto-parietal Task Control | 4 | 0.30 |
| 171 | frontal_pole/MFG | Fronto-parietal Task Control | 0 | 0.00 |
| 172 | frontal_pole/IFG | Fronto-parietal Task Control | 0 | 0.00 |
| 173 | SPL/IPL | Fronto-parietal Task Control | 2 | 0.35 |
| 174 | frontal_pole | Fronto-parietal Task Control | 0 | 0.00 |
| 175 | MFG | Fronto-parietal Task Control | 0 | 0.00 |
| 176 | mFC/paracingulate | Fronto-parietal Task Control | 0 | 0.00 |
| 177 | precuneus | Salience | 2 | 0.35 |
| 178 | SMG | Salience | 0 | 0.00 |
| 179 | precentral_gyrus | Salience | 0 | 0.00 |
| 180 | MFG | Salience | 0 | 0.00 |
| 181 | IFG | Salience | 1 | 0.31 |
| 182 | insula | Salience | 1 | 0.25 |
| 183 | insula | Salience | 1 | 0.26 |
| 184 | insula | Salience | 1 | 0.28 |
| 185 | insula | Salience | 4 | 0.39 |
| 186 | ACC | Salience | 0 | 0.00 |
| 187 | mFC/paracingulate | Salience | 1 | 0.26 |
| 188 | frontal_pole_sup/SFG | Salience | 0 | 0.00 |
| 189 | ACC | Salience | 1 | 0.28 |
| 190 | ACC | Salience | 3 | 0.29 |
| 191 | ACC | Salience | 1 | 0.31 |
| 192 | frontal_pole_sup/SFG | Salience | 2 | 0.34 |
| 193 | frontal_pole_sup/SFG | Salience | 1 | 0.26 |
| 194 | frontal_pole_sup/MFG | Salience | 2 | 0.31 |
| 195 | thalamus | Subcortical | 0 | 0.00 |
| 196 | thalamus | Subcortical | 1 | 0.29 |
| 197 | thalamus | Subcortical | 2 | 0.37 |
| 198 | thalamus | Subcortical | 4 | 0.38 |
| 199 | brainstem/SC | Subcortical | 0 | 0.00 |
| 200 | putamen | Subcortical | 0 | 0.00 |
| 201 | putamen/white_matter | Subcortical | 2 | 0.37 |
| 202 | putamen | Subcortical | 0 | 0.00 |
| 203 | putamen | Subcortical | 1 | 0.27 |
| 204 | putamen | Subcortical | 1 | 0.26 |
| 205 | putamen | Subcortical | 1 | 0.25 |
| 206 | putamen/white_matter | Subcortical | 4 | 0.38 |
| 207 | thalamus | Subcortical | 12 | 0.36 |
| 208 | SMG/STG/TPJ | Ventral attention | 2 | 0.39 |
| 209 | MTG | Ventral attention | 0 | 0.00 |
| 210 | STG | Ventral attention | 0 | 0.00 |
| 211 | STG | Ventral attention | 1 | 0.25 |
| 212 | MTG | Ventral attention | 0 | 0.00 |
| 213 | MTG/STG | Ventral attention | 2 | 0.34 |
| 214 | IFG | Ventral attention | 0 | 0.00 |
| 215 | IFG | Ventral attention | 1 | 0.27 |
| 216 | SPL | Dorsal attention | 5 | 0.40 |
| 217 | MTG/LOC | Dorsal attention | 0 | 0.00 |
| 218 | postcentral_gyrus/SMG_border | Sensory/somatomotor Hand | 0 | 0.00 |
| 219 | SPL | Dorsal attention | 0 | 0.00 |
| 220 | MTG/LOC | Dorsal attention | 1 | 0.26 |
| 221 | SPL | Dorsal attention | 0 | 0.00 |
| 222 | SPL | Dorsal attention | 0 | 0.00 |
| 223 | LOC/parietal_border | Dorsal attention | 0 | 0.00 |
| 224 | MFG | Dorsal attention | 0 | 0.00 |
| 225 | ITG/fusiform | Dorsal attention | 0 | 0.00 |
| 226 | SPL | Dorsal attention | 3 | 0.36 |
| 227 | precentral_gyrus | Dorsal attention | 0 | 0.00 |

**Table S5. The group difference of REM connectome**

| Network Num. | F | P | P-fdr |
| --- | --- | --- | --- |
| 1 | 1.06 | 0.35 | 0.46 |
| 2 | 2.21 | 0.12 | 0.25 |
| 3 | 0.59 | 0.56 | 0.62 |
| 4 | 0.10 | 0.90 | 0.93 |
| 5 | 4.39 | 0.01 | 0.12 |
| 6 | 3.73 | 0.03 | 0.14 |
| 7 | 3.63 | 0.03 | 0.14 |
| 8 | 5.04 | 0.01 | 0.09 |
| 9 | 1.00 | 0.37 | 0.46 |
| 10 | 0.81 | 0.45 | 0.53 |
| 11 | 2.23 | 0.11 | 0.25 |
| 12 | 1.71 | 0.19 | 0.33 |
| 13 | 0.35 | 0.71 | 0.76 |
| 14 | 2.48 | 0.09 | 0.22 |
| 15 | 8.10 | 0.00 | 0.02 |
| 16 | 2.93 | 0.06 | 0.18 |
| 17 | 4.05 | 0.02 | 0.13 |
| 18 | 1.66 | 0.20 | 0.33 |
| 19 | 1.04 | 0.36 | 0.46 |
| 20 | 1.16 | 0.32 | 0.46 |
| 21 | 3.20 | 0.04 | 0.16 |
| 22 | 6.19 | 0.00 | 0.05 |
| 23 | 1.10 | 0.34 | 0.46 |
| 24 | 2.61 | 0.08 | 0.21 |
| 25 | 3.17 | 0.05 | 0.16 |
| 26 | 1.32 | 0.27 | 0.42 |
| 27 | 2.69 | 0.07 | 0.21 |
| 28 | 1.81 | 0.17 | 0.32 |
| 29 | 0.02 | 0.98 | 0.98 |
| 30 | 0.65 | 0.52 | 0.60 |
| 31 | 1.60 | 0.21 | 0.33 |
| 32 | 2.00 | 0.14 | 0.28 |

|  | SMN | CON | AUD | DMN | VIS | FPN | SAN | SUN | VAN | DAN |
| --- | --- | --- | --- | --- | --- | --- | --- | --- | --- | --- |
| SMN | 0 | 0 | 0 | 0 | 0 | 0 | 0 | 0 | 0 | 0 |
| CON | 1 | 7 | 0 | 0 | 0 | 0 | 0 | 0 | 0 | 0 |
| AUD | 2 | 8 | 0 | 0 | 0 | 0 | 0 | 0 | 0 | 0 |
| DMN | 3 | 0 | 0 | 15 | 0 | 0 | 0 | 0 | 0 | 0 |
| VIS | 0 | 0 | 12 | 16 | 19 | 0 | 0 | 0 | 0 | 0 |
| FPN | 0 | 0 | 0 | 0 | 20 | 24 | 0 | 0 | 0 | 0 |
| SAN | 4 | 9 | 13 | 0 | 21 | 25 | 28 | 0 | 0 | 0 |
| SUN | 5 | 10 | 0 | 17 | 22 | 26 | 29 | 0 | 0 | 0 |
| VAN | 6 | 0 | 0 | 0 | 23 | 27 | 0 | 31 | 0 | 0 |
| DAN | 0 | 11 | 14 | 18 | 0 | 0 | 30 | 0 | 0 | 32 |

**Network ID in Table S5**

**Table S6. The group difference of DMN-DMN connection**

| FC-Num. | F | P | ROI & ROI |
| --- | --- | --- | --- |
| 1 | 4.01 | 0.02 | mFC/paracingulate&mFC/paracingulate |
| 2 | 3.11 | 0.05 | frontal_pole_sup/SFG&precuneus/PCC |
| 3 | 1.80 | 0.17 | frontal_pole_sup/SFG&PCC |
| 4 | 1.24 | 0.29 | fusiform&frontal_pole_sup/SFG |
| 5 | 3.11 | 0.05 | precuneus/PCC&frontal_pole_sup/SFG |
| 6 | 1.80 | 0.17 | PCC&frontal_pole_sup/SFG |
| 7 | 2.56 | 0.08 | mFC/paracingulate&frontal_pole_sup/SFG |
| 8 | 2.36 | 0.10 | mFC/paracingulate&frontal_pole_sup/SFG |
| 9 | 4.04 | 0.02 | MTG/ITG&frontal_pole_sup/SFG |
| 10 | 2.57 | 0.08 | cerebellum&frontal_pole_sup/SFG |
| 11 | 2.56 | 0.08 | frontal_pole_sup/SFG&mFC/paracingulate |
| 12 | 4.01 | 0.02 | mFC/paracingulate&mFC/paracingulate |
| 13 | 2.36 | 0.10 | frontal_pole_sup/SFG&mFC/paracingulate |
| 14 | 4.04 | 0.02 | frontal_pole_sup/SFG&MTG/ITG |
| 15 | 1.24 | 0.29 | frontal_pole_sup/SFG&fusiform |
| 16 | 2.57 | 0.08 | frontal_pole_sup/SFG&cerebellum |

**TableS7. The post-hoc analysis between groups of DMN-DMN connection**

| FC Num. | Late Sleep VS. Early Dep. | | Late Sleep VS. Late Dep. | | Early Dep.VS. Late Dep. | | ROI & ROI |
| --- | --- | --- | --- | --- | --- | --- | --- |
|  | T | P | T | P | T | P |  |
| 1 | 0.99 | 0.33 | 2.558866 | 0.01 | 2.05 | 0.043405997 | mFC/paracingulate&mFC/paracingulate |
| 2 | 0.29 | 0.78 | 2.212986 | 0.03 | 2.15 | 0.034719269 | frontal_pole_sup/SFG&precuneus/PCC |
| 3 | 0.29 | 0.78 | 2.212986 | 0.03 | 2.15 | 0.034719269 | precuneus/PCC&frontal_pole_sup/SFG |
| 4 | -0.83 | 0.41 | 1.937212 | 0.06 | 2.66 | 0.009493262 | MTG/ITG&frontal_pole_sup/SFG |
| 5 | 0.99 | 0.33 | 2.558866 | 0.01 | 2.05 | 0.043405997 | mFC/paracingulate&mFC/paracingulate |
| 6 | -0.83 | 0.41 | 1.937212 | 0.06 | 2.66 | 0.009493262 | frontal_pole_sup/SFG&MTG/ITG |

**Table S8. The group difference of Visual-Subcortex connection**

| FC-Num. | F | P | ROI & ROI |
| --- | --- | --- | --- |
| 1 | 4.09 | 0.019335 | occip_cortex/intracalcarine&thalamus |
| 2 | 2.87 | 0.06135 | lingual_gyrus&thalamus |
| 3 | 2.80 | 0.065451 | occip_cortex/intracalcarine&putamen/white_matter |
| 4 | 3.22 | 0.043671 | occip_cortex/intracalcarine&putamen/white_matter |
| 5 | 6.51 | 0.002145 | occip_cortex/intracalcarine&thalamus |
| 6 | 5.91 | 0.003661 | occip_cortex/intracalcarine&thalamus |
| 7 | 4.28 | 0.016283 | lingual_gyrus&thalamus |
| 8 | 6.34 | 0.002504 | occip_cortex/intracalcarine&thalamus |
| 9 | 2.04 | 0.135327 | occip_cortex/fusiform&thalamus |
| 10 | 7.69 | 0.000761 | lingual_gyrus&thalamus |
| 11 | 4.31 | 0.015862 | occip_cortex/intracalcarine&thalamus |

**TableS9. The post-hoc analysis between groups of Vis-Sub connection**

| FC Num. | Late Sleep VS. Early Dep. | | Late Sleep VS. Late Dep. | | Early Dep.VS. Late Dep. | | ROI & ROI |
| --- | --- | --- | --- | --- | --- | --- | --- |
|  | T | P | T | P | T | P |  |
| 1 | 2.68 | 0.01 | 2.16 | 0.03 | -0.39 | 0.70 | occip_cortex/intracalcarine&thalamus |
| 2 | 1.41 | 0.16 | 2.52 | 0.01 | 1.23 | 0.22 | occip_cortex/intracalcarine&putamen/white_matter |
| 3 | 2.98 | 0.00 | 3.42 | 0.00 | 0.69 | 0.49 | occip_cortex/intracalcarine&thalamus |
| 4 | 3.13 | 0.00 | 3.04 | 0.00 | 0.11 | 0.91 | occip_cortex/intracalcarine&thalamus |
| 5 | 2.63 | 0.01 | 2.62 | 0.01 | 0.13 | 0.90 | lingual_gyrus&thalamus |
| 6 | 2.46 | 0.02 | 3.69 | 0.00 | 1.13 | 0.26 | occip_cortex/intracalcarine&thalamus |
| 7 | 2.69 | 0.01 | 4.00 | 0.00 | 1.39 | 0.17 | lingual_gyrus&thalamus |
| 8 | 1.88 | 0.06 | 2.99 | 0.00 | 1.14 | 0.26 | occip_cortex/intracalcarine&thalamus |
